# Immuno-metabolic signaling in leishmaniasis: insights gained from mathematical modeling

**DOI:** 10.1093/bioadv/vbad125

**Published:** 2023-09-14

**Authors:** Shweta Khandibharad, Shailza Singh

**Affiliations:** Systems Medicine Laboratory, National Centre for Cell Science, NCCS Complex, SPPU Campus, Pune 411007, India; Systems Medicine Laboratory, National Centre for Cell Science, NCCS Complex, SPPU Campus, Pune 411007, India

## Abstract

**Motivation:**

Leishmaniasis is a global concern especially in underdeveloped and developing subtropical and tropical regions. The extent of infectivity in host is majorly dependent on functional polarization of macrophages. Classically activated M1 macrophage can eliminate parasite through production of iNOS and alternatively activated M2 macrophages can promote parasite growth through by providing shelter and nutrients to parasite. The biological processes involved in immune signaling and metabolism of host and parasite might be responsible for deciding fate of parasite.

**Results:**

Using systems biology approach, we constructed two mathematical models and inter-regulatory immune-metabolic networks of M1 and M2 state, through which we identified crucial components that are associated with these phenotypes. We also demonstrated how parasite may modulate M1 phenotype for its growth and proliferation and transition to M2 state. Through our previous findings as well as from recent findings we could identify SHP-1 as a key component in regulating the immune-metabolic characterization of M2 macrophage. By targeting SHP-1 at cellular level, it might be possible to modulate immuno-metabolic mechanism and thereby control parasite survival.

**Availability and implementation:**

Mathematical modeling is implemented as a workflow and the models are deposited in BioModel database. FactoMineR is available at: https://github.com/cran/FactoMineR/tree/master.

## Graphical abstract

**Graphical abstract:**
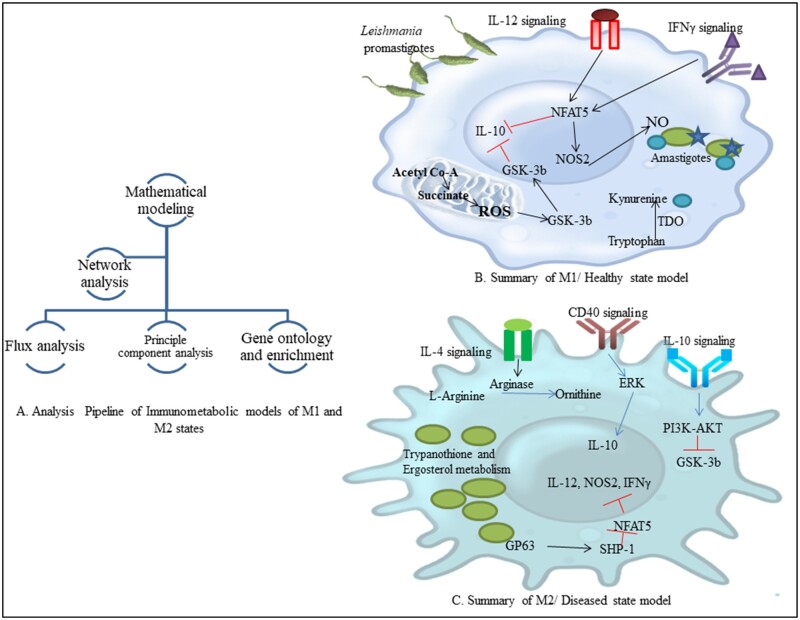

## 1 Introduction

Leishmaniasis is a vector-borne disease caused by the protozoan parasite *Leishmania* (Steverding 2017). These parasites are digenetic and dimorphic in nature living inside the mammalian host. This disease is one of the major tropical and subtropical diseases. It is mainly transmitted by female sand flies of *Phlebotomus spp*. and *Lutzomyia spp*. by bite and blood meal. Leishmaniasis has been classified as an endemic across the world affecting Asia, Africa, the Americas, and the Mediterranean region (Torres-Guerrero *et al.* 2017). States that are majorly affected in India are Bihar, Kerala, Madhya Pradesh, Haryana, Uttarakhand, Himachal Pradesh, Assam, Rajasthan, Uttar Pradesh, Jammu and Kashmir, Punjab, and Delhi (Thakur *et al.* 2018, Kumar *et al.* 2020).

Visceral (VL), mucocutaneous (ML), or cutaneous leishmaniasis (CL) are the three different clinical manifestations of leishmaniasis. The World Health Organization (WHO) estimates that in 2020, VL was the primary form in more than 90% of cases that occurred in Brazil, Ethiopia, India, Kenya, Somalia, South Sudan, and Sudan. In 10 nations—Afghanistan, Algeria, Brazil, Colombia, Iraq, Pakistan, Peru, Syria Arab Republic, Tunisia, and Yemen—more than 84% of the cases recorded in 2016 were due to CL (Scarpini *et al.* 2022). There are numerous distinct *Leishmania* parasites that cause leishmaniasis in the Middle East. CL is the most reported form of the disease which is identified by ulcerative skin lesions. Due to its propensity for self-healing and rarity of fatalities, CL is an extremely neglected form of leishmaniasis. *Leishmania mexicanas* and *Leishmania braziliensis* complex are more prevalent in the New World whereas *Leishmania tropica, Leishmania major* and *Leishmania aethiopica* are in the Old World (Thakur *et al.* 2018).

The life cycle of *Leishmania* involves two different stages. First cycle occurs in the female sandfly and second in the mammalian host (human or dogs). When a sandfly harboring *Leishmania* protozoa takes a blood meal, it injects saliva that prevents blood clotting (Ribeiro *et al.* 1986). Metacyclic promastigotes are released after blood ingestion at the bite site. Promastigotes are elongated and slender in shape, motile and present outside the cell. The first cells to be drawn to the biting site are neutrophils. These promastigotes are swallowed by neutrophils, but since they are short-lived and go through apoptosis, it has been suggested that they serve as “Trojan horses” that parasites utilize to infiltrate macrophages and prevent cell activation (Regli *et al.* 2017). They divide and change into amastigotes inside the macrophage, which then infect the nearby tissues. These amastigotes are non-motile, small, and circular. When the sand fly consumes a blood meal from the infected person, the infected macrophages are also taken up and burst in the sand fly’s midgut, releasing amastigotes. They later develop and migrate to the proboscis, where they can infect another individual (Leta *et al.* 2014).

At the site of infection, macrophages exhibit significant plasticity in response to converging signals from numerous factors such as chemokines, cytokines and growth factors present in the local tissue environment. Following that, macrophages would adopt a range of various polarization states, among which the M1 macrophage (inflammatory) and the M2 macrophage (anti-inflammatory) are the primary (Xue *et al.* 2014). Metabolic reprogramming regulates the polarization of macrophages. The metabolism of activated immune cells operates as a switch to regulate their immunological response in addition to providing their biosynthetic and bioenergetics requirements (Ty *et al.* 2019).

The M1 polarization is important for the control of infection by *Leishmania spp.* They are characterized by the impaired tricarboxylic cycle (TCA cycle), production of pro-inflammatory cytokines such as Interleukin-12 (IL-12), interferon gamma (IFNℽ) and by generation of reactive oxygen species (ROS) and nitric oxide (NO) that help in efficient parasite killing. The M2 macrophages are identified by intact TCA cycle, production of anti-inflammatory cytokines such as Interleukin-4 (IL-4), Interleukin-10 (IL-10) etc., which promote tissue repair and parasite survival (Wilson *et al.* 2019). Macrophage polarization is highly associated with changes in cellular metabolism and metabolic signaling pathways which support several essential macrophage functions, such as phagocytosis, motility, and mediator synthesis (NO, ROS, cytokines, and chemokines) (Srivastava *et al.* 2016, Saunders and McConville 2020). Alterations in intracellular metabolite concentrations may also directly influence signaling transduction pathways like low cholesterol level alters cluster of differentiation 40 (CD40) mediated IL-12 induction (Rub *et al.* 2009) and activate long-term epigenetic and transcriptional programs that promote M1 and M2 polarization. Hence, it is crucial to study immune-metabolic response in a holistic way to understand the changes in the macrophage polarization and metabolism that are induced by innate and adaptive immune responses which determine the outcome of infection.

Systems biology is an interdisciplinary field that analyses biological systems holistically by treating genes, proteins, biochemical networks, and physiological responses as integral components of a large system (Chuang *et al.* 2010). Being a holistic approach, systems biology has been used to investigate various diseases, by using both independent patient samples and publicly accessible datasets. System biology approach gives us the versatility that may be used for studying the immuno-metabolism characterization of macrophages associated with *Leishmania* infection which may help us to find potential key component whose modulation can be used in parasite clearance and restore balance.

In M1 model, we presented the initial stage of infection where the host immuno-metabolic response is working against the parasite. It initiates when lipophosphoglycan (LPG) of parasite interacts with toll-like receptors (TLRs), TLR2 and TLR6, that activate myeloid differentiation primary response 88 (MyD88) which promotes nuclear factor kappa B (NFκB) activation and IL-12 synthesis. Crucial transcription factors, including interferon regulatory factor 1 (IRF1), c-Rel and nuclear factor of activated T cells 5 (NFAT5), are involved in the synthesis of IL-12 (Ma *et al*. 2015). NFAT5 can not only upregulate IL-12, but also induce IFNγ, inducible nitric oxide synthase (iNOS) production and inhibit IL-10 transcription (Huerga Encabo *et al.* 2020, Khandibharad and Singh, 2022a). IFNγ signaling is known to be induced by IL-12 via the Janus kinase/signal transducers and activators of transcription (JAK-STAT) signaling that induces STAT4 mediated pathway (Liu *et al.* 2005). IFNγ further induces JAK-STAT1-mediated signaling cascade and initiates NO production (Rosas *et al.* 2003). Simultaneously, glycolytic activity is enhanced and then Acetyl CoA is directed to impaired TCA cycle in which flux through succinate dehydrogenase is blocked causing succinate accumulation(Liu *et al.* 2021). Succinate dehydrogenase (SDH) acts as electron transport chain (ETC) complex-II which may lead to succinate oxidation; producing a high proton motive force which may promote reverse electronic transmission (RET) in the complex-I, generating mitochondrial ROS (mtROS) (Mills *et al.* 2016, Volpedo *et al.* 2022). *Leishmania* growth inside host macrophage may also require amino acids like tryptophan. In M1 macrophage, the upregulation of tryptophan 2, 3- deoxygenase (TDO) convert tryptophan to kynurenine, reducing the availability of tryptophan to amastigotes. TDO levels are negatively correlated with parasite load (Rodrigues *et al.* 2019). Macrophages also utilize tryptophan for NAD+ biosynthesis which further stimulates the regeneration of ROS from complex 3 of ETC (Billingham and Chandel 2019). Later, ROS plays a major role in the activation of glycogen synthase kinase-3 beta (GSK-3b) (Wang *et al.* 2009), which suppresses the AP-1 function by suppressing DNA binding, resulting in decreased transcription of IL-10 (Cortés-Vieyra *et al.* 2021).

In the M2 model, we presented an infection condition in which intracellular amastigotes alter the immuno-metabolic responses to help in their survival and proliferation. It starts with LPG interaction with TLR2 and TLR6, which leads to IL-10 production mediated by p50 dimer NFκB (Guizani-Tabbane *et al.* 2004). This interaction also initiates phosphoinositide 3-kinase (PI3K) mediated GSK-3b inhibition through Protein kinase B (AKT) pathway (Panigrahy *et al.* 2020). IL-4 also contributes to M2 polarization by inducing Arginase 1 (Arg1). Arg1 is a cytosolic enzyme that converts L-arginine into urea and ornithine, which are precursors of polyamines that promote parasite proliferation and production of trypanothione, a dithiol necessary for parasite defence against oxidants (NO and ROS). In addition, Arg1 also competes with NOS2 for the same substrate, L-arginine, which prevents NO synthesis (Bogdan 2020). *Leishmania* also secretes some proteases like GP63. GP63 activates host protein tyrosine phosphatase, non-receptor type 6 (SHP-1) that later inhibits NFAT5 and prevents IL-12, IFNγ, and NO production (Shio and Olivier 2010). GP63 also contributes to cholesterol depletion, which leads to CD40 signaling reciprocity. Instead of following the p38k mediated IL-12 production pathway, it follows extracellular signal-regulated protein kinases 1 and 2 (ERK1/2) mediated IL-10 production (Descoteaux *et al.* 2013). Amastigotes may exchange host cholesterol for ergosterol, which is used for its membrane that acts as the first defense inside host macrophage (Rub *et al.* 2009). SHP-1 also plays a vital role in CD40 signaling reciprocity, governing IL-12 and IL-10 production (Khan *et al.* 2014).

Using these mathematical models and immune-metabolic networks, we identified that SHP-1 can be an important factor that controls the immune-metabolic characterization of macrophage as it inhibits NFAT5, which regulates cytokines’ synthesis like IL-12, IFNγ, and IL-10 as well as NO production. SHP-1 also regulates metabolic processes like glucose homeostasis (Dubois *et al.* 2006). Literature reports experimentally that increased SHP-1 activity is observed in *Leishmania*-infected macrophages and its inhibition reverses the phenotype of macrophages to eliminate the parasite (Nandan *et al.* 2012). Reduced expression of SHP-1 has been reported to resemble reduced M2 phenotype as compared to wild type macrophages (Myers *et al.* 2020). Thus, its presence or absence can play a significant role in determining the characterization of macrophage and the fate of the disease. We also present SHP-1 as a potential target whose inhibition can lead to parasite clearance.

## 2 Methods

### 2.1 Reconstruction of model and its analysis

In this study, two mathematical models were reconstructed; one showing the parasite killing phenotype of macrophages through expressing the pro-inflammatory cytokines like IL-12, IFNℽ as well as NO and ROS (healthy state) and the other functioning as the early stage of M1 to M2 polarization in which the macrophage resident amastigotes leads to IL-10 expression and also use host immuno-metabolic pathways to produce Arg1, trypanothione, and ergosterol for their survival and proliferation (diseased state).

Both models were reconstructed in Cell Designer and exported as systems biology markup Language (SBML) file. The biological reactions in the model were mathematically defined and quantified by including the kinetics rate law, parameter estimation, initial component concentration (Kumar *et al.* 2020). The kinetic rate laws which are used to define reactions of the reconstructed models in the Cell designer were law of mass action (association, dissociation, transcription, translation, activation), Michaelis–Menten equation (enzyme-involved reactions), and non-competitive inhibition (inhibitory reaction).

By considering experimentally known concentrations from the literature which suggests that a cell can secrete 10^3^–10^6^ signaling molecules, initial concentration of the components was defined (Moran *et al.* 2010, Khandibharad and Singh, 2022a).

By estimating the parameters, the model equation was chosen in such a way that made the mathematical model resemble the behavior of our previous findings (Khandibharad and Singh, 2022a, 2022b). Mathematical simulation of the defined reactions was done using SOSlib solver (1.7.0).

### 2.2 Flux analysis

Flux analysis is a constraint-based method that attempts to establish a phenotype for the reactions in a particular biological system by analyzing the steady-state flow distribution. It is predicated on the idea that all expressed phenotypes in a particular biological system must adhere to fundamental constraints imposed on all cell activities (Min Lee *et al.* 2008).

The flux was calculated using COPASI (4.36.260), a biochemical network simulator. It solves mathematical model ODEs and determines the flux of each reaction in the reconstructed biological network. The biological system’s outcome is determined by the rate at which components are produced, hence a reaction with a higher flux is crucial in the network (Jawale *et al.* 2023). To filter out high flux reactions, we have taken the threshold of approximately five times of the lowest flux reaction.

### 2.3 Principal component analysis (PCA)

Principal component analysis (PCA) is a multivariate approach for analyzing a data table in which observations are characterized by numerous inter-correlated quantitative dependent variables. Its intent is to obtain important information from the data table and represent it as a set of new variables known as principal components. PCA also depicts the similarity pattern of the data and variables by presenting them as points on maps (Abdi and Williams, 2010). We used Factoextra (4.1.3) and FactoMineR (4.1.3) packages to perform PCA in R studio (4.1.1).

The PCA input file contains information regarding each of the components present in M1 and M2, such as the name of the model in which that component is present, as well as the concentration of production/utilization.

### 2.4 Network construction and analysis

Cytoscape is an open-source software platform for building molecular interaction networks and biological pathways and integrating these networks with annotations, gene expression profiles and other state data. It also provides different plug-ins for the analysis of our network (Shannon *et al.* 2003).

For the evaluation of the networks CytoHubba plugin was used. The top ten nodes in CytoHubba were selected depending on all the topological analysis methods including degree, edge percolated component (EPC), maximum neighbourhood component (MNC), density of maximum neighbourhood component (DMNC), maximal clique centrality (MCC) and six centralities (bottleneck, eccentricity, closeness, radiality, betweenness, and stress) based on shortest paths.

The Cytoscape plugin program Biological Networks Gene Ontology tool (BiNGO, v3.0.5) collected GO annotations for the M1 and M2 components. BiNGO retrieved the overrepresentation of GO categories in a sub-graph of a biological network, which is displayed on Cytoscape, using the input list of M1 and M2 components. The Benjamini and Hochberg correction was used to give tight control over the false discovery rate under positive regression dependence of the test statistics, and the hyper geometric test *P*-value was adjusted at 0.01.

## 3 Results

### 3.1 Mathematical models and simulation

#### 3.1.1 M1 model

The healthy state mathematical model had four compartments including cell membrane, cytoplasm, mitochondria and nucleus. A total of 97 components and 74 reactions were used to construct the model. Simulation for 20 time units was performed, using SBML ODE Solver Library SOSlib (1.7.0) available in Cell Designer (4.4.2).

**Figure 1.**
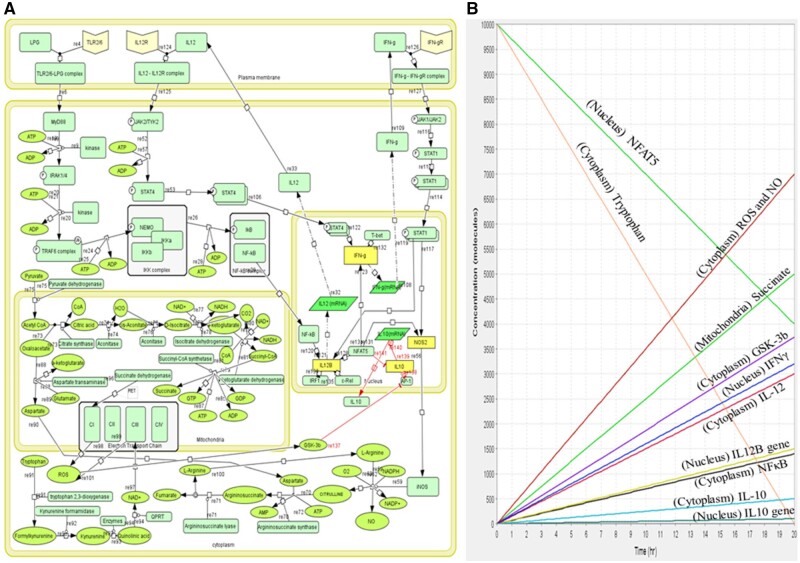
(A) Mathematical model of healthy state showing M1 phenotype, where immuno-metabolic response is leading to parasite clearance. (B) Simulation of M1 model showing concentration of pro-inflammatory cytokines, transcription factor (NFAT5) and metabolites for 20 time units.

In the M1 model, we observed that LPG bound to TLRs activated MyD88-mediated signaling, which in turn triggered the transcription of IL-12 through NFκB and lead to the activation of IFNγ, which further produced NO. Succinate dehydrogenase participated in the formation of ROS through ETC when the Krebs cycle was simultaneously hindered, which causes succinate accumulation. In addition, tryptophan was being catabolized in order to use it for the formation of ROS and prevent the parasite from using it as a nutrient source. Moreover, ROS also activated GSK-3b, which in turn prevented IL-10 from being transcribed. These combined processes are what allow for the elimination of parasites (Fig. 1A). We observed concentration of these prime markers, such as NFAT5, tryptophan, ROS, NO, GSK-3b, IL-12, and IFNγ, were high and the concentrations of IL-10 were low, maintaining the M1 state (Fig. 1B). The model is deposited in BioModel database (Malik-Sheriff *et al.* 2020) with the identifier as MODEL2305300001. ODEs for crucial reactions are enlisted in Table 1.

**Table 1.** Reactions and ordinary differential equations (ODEs) of simulated components from healthy state (M1 model).

Reactions	Ordinary differential equations
Nf-kB -> IL 12B	d [IL 12B]/d[t] = k* [Nf-kB]
IL 12B -> IL 12 (mRNA)	d [IL 12 (mRNA)]/d[t] = k* [IL 12B]
Nf-kB complex -> Nf-kB	d [Nf-kB]/d[t] = k* [Nf-kB complex]
NFAT5 -> IL12B	d [IL12B]/d[t] = k* [NFAT5]
STAT4(Nucleus) -> IFN-g	d [IFN-g]/d[t] = k* [STAT4(Nucleus)]
NFAT5 -> N0S2	d [N0S2]/d[t] = k* [NFAT5]
Electron Transport Chain -> ROS	d [ROS]/d[t] = k* [Electron Transport Chain]
Tryptophan -> Formylkynurenine; tryptophan 2,3-dioxygenase	d[Formylkynurenine]/d[t] = k* [Tryptophan]
Succinyl-CoA -> Succinate; “Succinyl-CoA synthetase”	d[Succinate]/d[t] = k* [Succinyl-CoA]
ROS -> GSK-3b	d[GSK-3b]/d[t] = k* [ROS]
IL10 -> “IL 10(mRNA)”	d[IL 10(mRNA)]/d[t] = k* [IL10]
IL 10(mRNA) -> “IL 10”	d[IL 10]/d[t] = k* [IL10(mRNA)]/]
NFAT5 -> IL10	d[IL-10]/d[t] = k* [NFAT5]

#### 3.1.2 M2 model

The diseased state mathematical model had four components which included cell membrane, cytoplasm, and nucleus and parasite cell. Model consists of 103 components and 85 reactions. The simulation was performed for 100 time units, using SBML ODE Solver Library SOSlib (1.7.0) available in Cell Designer.

**Figure 2.**
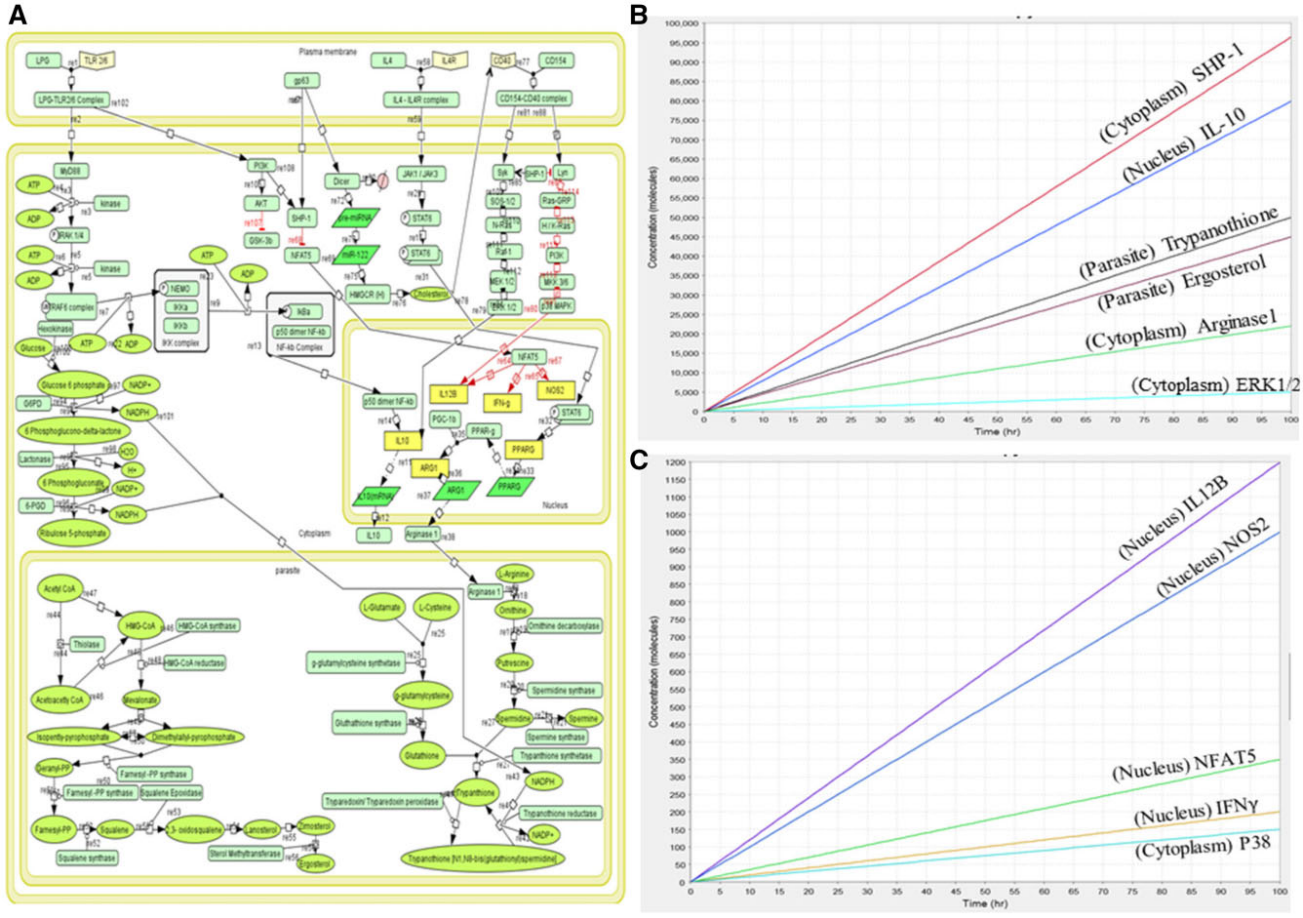
(A) Mathematical model of diseased state showing M2 phenotype, where immuno-metabolic response is leading to parasite survival. (B) Simulation of M2 model showing concentration of anti-inflammatory cytokines (IL-10), regulating factors (SHP-1, CD40, ERK 1/2), metabolites (Ergosterol, Trypanothione) and Arg1 for 100 time unit. (C) Simulation of M2 model showing reduction in concentration of pro-inflammatory cytokines, NFAT5, NOS2, p38 MAPK, and cholesterol.

In the M2 model, we found that the concentration of IL-10, ergosterol, and trypanothione had increased which showed parasite survival state (Fig. 2A). Concurrently due to cleavage of dicer by GP63, the cholesterol depletion occurs which leads to CD40 mediated IL-10 activation. GP63, also known to activate SHP-1 inhibited the expression of NFAT5 which blocks NO production. This observation from our model was previously reported by Forget *et al.* (2005). Due to which IL-12, IFNγ, and NOS2 expression had decreased thus leading to parasite survival (Fig. 2C). We observed that the main outputs of the system were the production of SHP-1, IL-10, Arg1, ergosterol, and trypanothione (Fig. 2B) with decreased concentration of IL-12, NFAT5, NOS2, and IFNγ (Fig. 2C). The model is deposited in BioModel database (Malik-Sheriff *et al.* 2020) with the identifier as MODEL2305300002. ODEs for crucial reactions are enlisted in Table 2.

**Table 2.** Reactions and ordinary differential equations (ODEs) of simulated components from diseased state (M2 model).

Reactions	Ordinary differential equations
gp63 -> SHP-1	d[SHP-1]/d[t] = k* [gp63]
IL 10(Nucleus) -> IL10 (mRNA)	d[IL10 (mRNA)]/d[t] = k* [IL 10(Nucleus)]
IL 10(mRNA) -> IL10 (cytoplasm)	d[IL10 (cytoplasm)]/d[t] = k* [IL 10(mRNA)]
Glutathione + Spermidine -> Trypanothione; Trypanothione synthetase	d[Trypanothione]/d[t] = k*[Glutathione] + [Spermidine]
Zimosterol -> Ergosterol; Sterol Methyltransferase	d[Ergosterol]/d[t] = k* [Zimosterol]
L-Arginine -> Ornithine; Arginase 1	d[Arginase 1]/d[t] = k* [L-Arginine]
MEK-1/2 -> ERK-1/2	d[ERK-1/2]/d[t] = k* [MEK-1/2]
NFAT5 -> IL12B	d[IL12B]/d[t] = k* [NFAT5]
NFAT5{Nucleus} -> NOS2	d[NOS2]/d[t] = k* [NFAT5{Nucleus}]
SHP-1 -> NFAT5{Cytoplasm}	d[NFAT5{Cytoplasm}]/d[t] = k* [SHP-1]
NFAT5{Nucleus} -> IFN-g	d[IFN-g]/d[t] = k* [NFAT5{Nucleus}]
MKK 3/6 -> “p38 MAPK”	d[p38 MAPK]/d[t] = k* [MKK 3/6]

### 3.2 Flux analysis

According to comparative flux analysis, the reaction associated with M1 model that had high flux are cytokines producing reaction such as IL-12, IFNγ, NOS2 activation, NO production, citrulline to L-arginine conversion, succinate accumulation through impaired Krebs cycle, tryptophan catabolism, ROS production, activation of GSK-3b. The flux value of each high flux reactions is mentioned in the table below (Table 3). Flux of all the reactions associated with M1 model is provided in Supplementary data (S1).

**Table 3.** High flux reaction of M1 model.

Reactions	Flux (mol/s)	Compartment
LPG + TLR 2/6 -> TLR2/6-LPG complex	499.5	Plasma membrane
TLR 2/6-LPG complex -> MyD88	449.91	Cytoplasm
MyD88 -> IRAK 1/4; Kinase	400	Cytoplasm
IRAK 1/4 -> TRAF 6 complex; Kinase	400	Cytoplasm
TRAF 6 complex -> IKK complex	369.926	Cytoplasm
IKK complex -> Nf-kB complex	369.926	Cytoplasm
Nf-kB complex -> Nf-kB	369.926	Nucleus
Nf-kB -> IL 12B	324.935	Nucleus
NFAT5 -> IL12B	99.98	Nucleus
IL 12B -> IL 12 (mRNA)	499.9	Nucleus
IL 12 (mRNA) -> IL12 (cytoplasm)	399.92	Cytoplasm
IL 12 (cytoplasm) -> IL 12 (Plasma membrane)	249.95	Plasma membrane
IL 12 (Plasma memnbrane) + IL12R -> IL12 - IL12R complex	499.5	Cytoplasm
IL 12 - IL12R complex -> JAK2/TYK2	449.91	Cytoplasm
JAK2/TYK2 -> STAT4 (cytoplasm)	349.93	Cytoplasm
2*STAT4 (cytoplasm) -> STAT4_2	124.975	Cytoplasm
STAT4(Nucleus) -> IFN-g(Nucleus)	99.98	Nucleus
NFAT5 -> IFN-g(Nucleus)	99.98	Nucleus
IFN-g (Plasma membrane) + IFN-gR -> IFN-g—IFN-gR complex	499.5	Plasma membrane
IFN-g -IFN-gR complex -> JAK1/JAK2	449.91	Cytoplasm
JAK1/JAK2 -> STAT1 (cytoplasm)	399.92	Cytoplasm
2 * STAT1(cytoplasm) -> STAT1_2	199.96	Cytoplasm
STAT1_2 -> STAT1(Nucleus)	99.98	Nucleus
NFAT5 -> N0S2	99.98	Nucleus
L-Arginine -> NO; iNOS	350	Cytoplasm
Aspartate (cytoplasm) + Citrulline -> Argininosuccinate; Argininosuccinate synthase	150	Cytoplasm
Argininosuccinate -> Fumarate + L-Arginine; Argininosuccinate lyase	100	Cytoplasm
Pyruvate -> Acetyl CoA; Pyruvate dehydrogenase	500	Cytoplasm
Acetyl CoA + Oxaloacetate -> Citric acid + CoA; Citrate synthase	450	Mitochondria
Citric acid -> cis-Aconitate + H2O; Aconitase	400	Mitochondria
cis-Aconitate + H2O -> D-isocitrate; Aconitase	400	Mitochondria
D-Isocitrate -> a-ketoglutarate + CO2; Isocitrate dehydrogenase	320	Mitochondria
a-ketoglutarate + CoA -> Succinyl-CoA + CO2; a-ketoglutarate dehydrogenase	300	Mitochondria
Succinyl-CoA -> Succinate; Succinyl-CoA synthase	250	Mitochondria
Succinate dehydrogenase -> Electron Transport Chain	249.95	Mitochondria
Tryptophan -> Formylkynurenine; tryptophan 2,3-dioxygenase	500	Cytoplasm
Formylkynurenine -> Kynurenine; Kynurenine formamidase	450	Cytoplasm
Kynurenine -> Quinolinic acid; Enzymes	400	Cytoplasm
Quinolinic acid -> NAD+ (cytoplasm); QPRT	400	Cytoplasm
NAD+ (cytoplasm) -> Electron Transport Chain	400	Mitochondria
Electron Transport Chain -> ROS	249.95	Cytoplasm
Electron Transport Chain -> ROS	299.94	Cytoplasm
ROS -> GSK-3b	199.96	Cytoplasm

The reactions associated with M2 model that had high flux were reactions that lead to production of IL10, polyamines production, trypanothione, and ergosterol. Activation of SHP-1, and AKT downstream pathway also had high flux. The flux value of each high flux reactions is mentioned in the table below (Table 4). Flux of all the reactions associated with M2 model is provided in Supplementary data (S2).

**Table 4.** High flux reaction of M2 model.

Reactions	Flux (mol/s)	Compartment
LPG + TLR 2/6 -> LPG-TLR 2/6 complex	999	Plasma Membrane
LPG-TLR 2/6 complex -> MyD88	979.02	Cytoplasm
MyD88 -> IRAK 1/4; kinase	900	Cytoplasm
IRAK 1/4 -> TRAF 6 complex; kinase	850	Cytoplasm
TRAF 6 complex -> IKK complex	799.2	Cytoplasm
IKK complex -> Nf-kb complex	699.3	Cytoplasm
IL 10(Nucleus) -> IL10 (mRNA)	999	Cytoplasm
IL 10(mRNA) -> IL10 (cytoplasm)	799.2	Cytoplasm
Nf-kb complex -> p50 dimer Nf-kb	649.935	Nucleus
p50 dimer Nf-kb -> IL 10 (Nucleus)	599.4	Nucleus
L-Arginine -> Ornithine; Arginase 1	1000	Parasite
Ornithine -> Putrescine; Ornithine decarboxylase	900	Parasite
Putrescine -> Spermidine; Spermidine synthase	800	Parasite
L-Glutamate + L-Cysteine -> g-Glutamylcysteine; ƴ-glutamylcysteine synthase	900	Parasite
g-Glutamylcysteine -> Glutathione; Gluthathione synthase	800	Parasite
Glutathione + Spermidine -> Trypanothione; Trypanothione synthetase	700	Parasite
Trypanothione -> Trypanothione [N1, N8-bis(glutathionyl) spermidine]; Tryparedoxin/Tryparedoxin peroxidase	700	Parasite
Trypanothione [N1, N8-bis(glutathionyl) spermidine] -> Trypanothione; Trypanothione reductase	500	Parasite
Acetyl CoA -> Acetoacetly CoA; Thiolase	500	Parasite
Acetoacetly CoA -> HMG-CoA; HMG-CoA synthase	450	Parasite
Acetyl CoA -> HMG-CoA	449.55	Parasite
HMG-CoA -> Mevalonate; HMG-CoA reductase	850	Parasite
Mevalonate -> Isopently-pyrophosphate + Dimethylallyl-pyrophosphate	799.2	Parasite
Isopently-pyrophosphate + Dimethylallyl-pyrophosphate -> Geranyl-PP; Farnesyl-PP synthase	700	Parasite
Geranyl-PP -> Farnesyl-PP; Farnesyl-PP synthase	650	Parasite
Farnesyl-PP -> Squalene; Squalene synthase	600	Parasite
Squalene -> 2,3- oxidosqualene; Squalene Epoxidase	550	Parasite
2,3- oxidosqualene -> Lanosterol	499.5	Parasite
Lanosterol -> Zimosterol	454.545	Parasite
Zimosterol -> Ergosterol; Sterol Methyltransferase	450	Parasite
IL4 + IL4R -> IL4 - IL4R complex	999.9	Plasma Membrane
IL4 - IL4R complex -> JAK1/JAK3	899.91	Cytoplasm
JAK1/JAK3 -> STAT6(cytoplasm)	849.15	Cytoplasm
gp63 -> SHP-1	799.2	Cytoplasm
CD154 + CD40 -> CD154-CD40 complex	999.9	Plasma Membrane
CD154-CD40 complex -> Syk	699.3	Cytoplasm
Syk -> SOS-1/2	679.32	Cytoplasm
SOS-1/2 -> N-Ras	649.35	Cytoplasm
N-Ras -> Raf-1	599.4	Cytoplasm
Raf-1 -> MEK-1/2	579.42	Cytoplasm
ERK 1/2 -> IL10 (Nucleus)	499.5	Nucleus
MEK-1/2 -> ERK-1/2	549.45	Cytoplasm
PI3K -> AKT	599.4	Cytoplasm

### 3.3 Principal component analysis (PCA)

**Figure 3.**
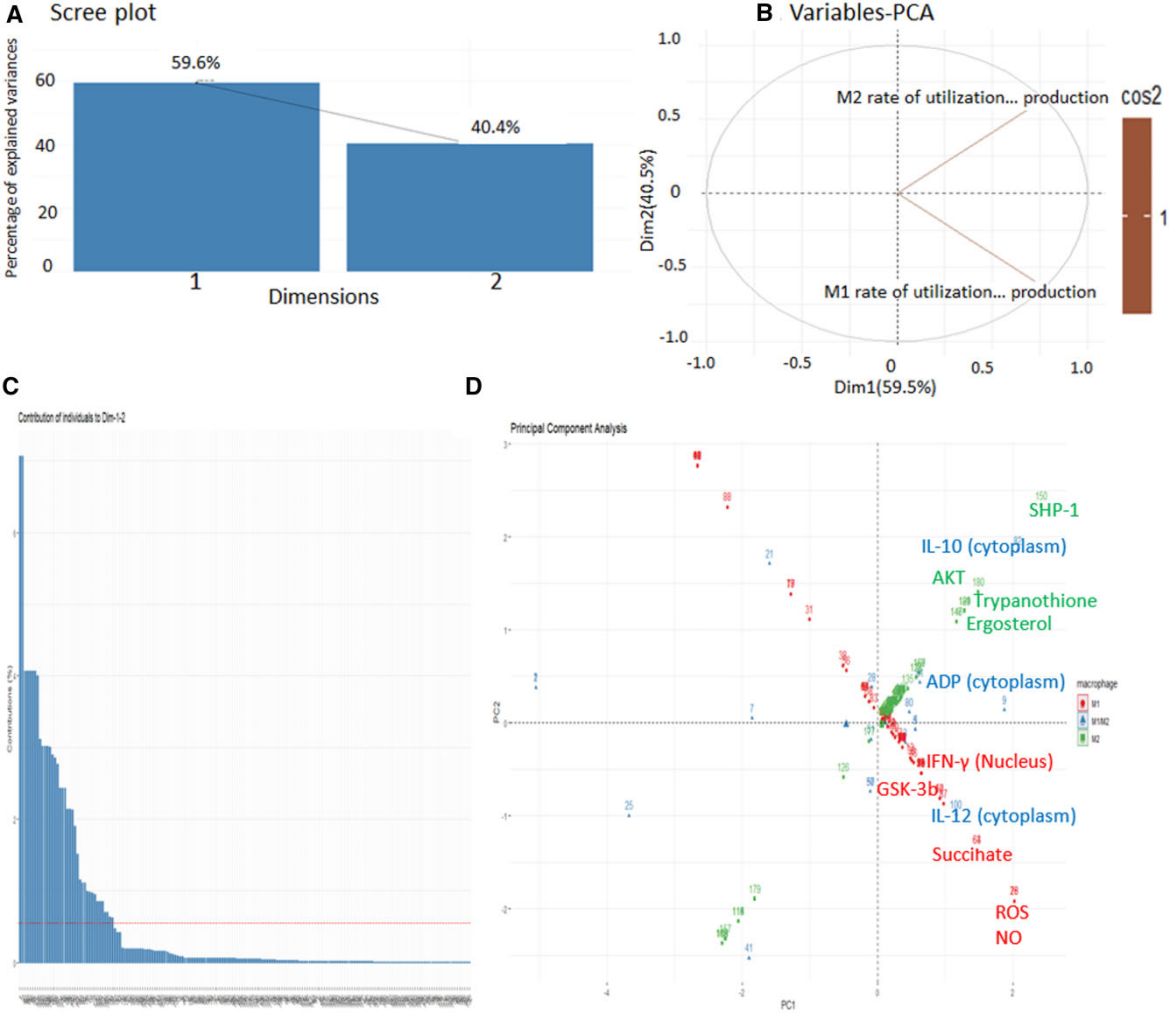
Principle component analysis (A) Scree plot (B) Variable plot showing the inverse correlation of M1 and M2. (C) Individual contribution of each component which is collectively present in both models. (D) PCA plot showing principal components.

With only two datasets, the scree plot does not show the elbow, which means that maybe both datasets are equally important. (Fig. 3A). Furthermore, in variable PCA plot, M1 and M2 dataset showed inverse correlation. The M2 components were in positive quadrant whereas the M1 components were in negative–positive quadrant, which state that the common component which were present in both M1 and M2 were negative due to low values, whereas the unique component of M1 is in positive (Fig. 3B). Moreover, the individual contribution graph worked as the first filter to select principal components, based on threshold score of 1. Total 38 components possessed PC score greater than 1 (Fig. 3C). To further filter out the principal component from 38 components, we identified PCs which are common as well as unique for M1 and M2 (Fig. 3D). Common components in both models after PCA were IL 10 (cytoplasm), GSK-3b, and ADP (cytoplasm). In addition to the common components, there were many other components that were model specific but equally important since PCA facilitates identification and increases the interpretability of critical components by minimizing information loss.

Unique components from M1 model which stood out from comparative analysis of both models were IFN-g (nucleus), IL12 (cytoplasm), succinate, ROS, and NO. In case of M2 model, they were SHP-1, AKT, trypanothione, and ergosterol (Fig. 3D). Matrix used for PCA is provided in Supplementary data (S3).

### 3.4 Network analysis

**Figure 4.**
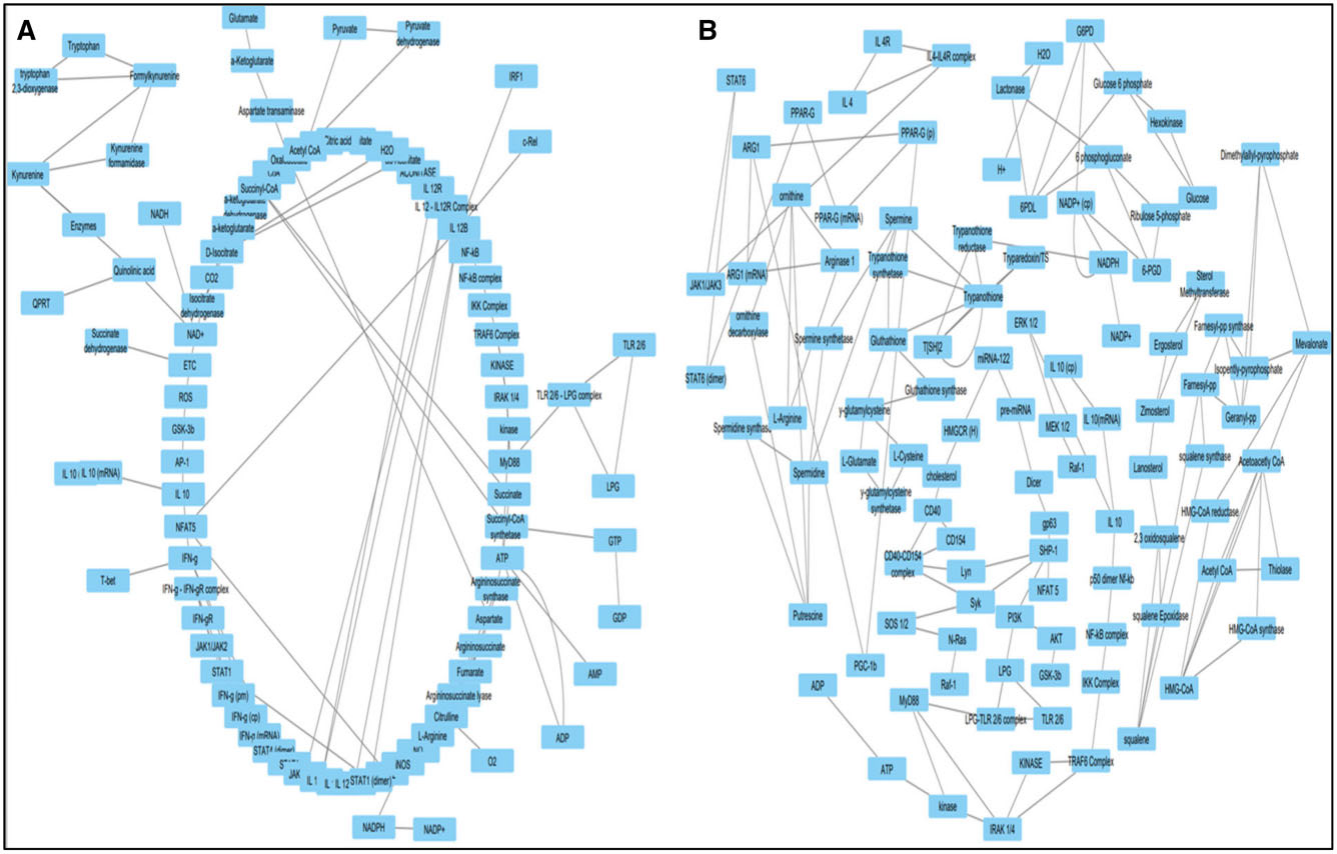
(A) Circular layout of M1 network and (B) Circular layout of M2 network.

Two networks were constructed based on the connections present between the components of M1 and M2 models (Fig. 4). The connections for M1 and M2 models is provided in Supplementary data (S4 and S5), respectively. Statistical analysis summary for both models is provided in Table 5.

**Table 5.** Network analysis shows the network’s resilience depending on various parameters.

Summary statistics of M1 network	
Number of nodes	97
Number of edges	127
Avg. number of neighbors	2.739
Network diameter	15
Network radius	10
Characteristics path length	6.918
Clustering coefficient	0.311
Network density	0.030
Network heterogeneity	0.493
Network centralization	0.037
Connected components	1
Summary statistics of M2 network	
Number of nodes	103
Number of edges	137
Avg. number of neighbors	2.696
Network diameter	23
Network radius	12
Characteristics path length	8.028
Clustering coefficient	0.415
Network density	0.060
Network heterogeneity	0.362
Network centralization	0.077
Connected components	3

The entire network was analysed using parameters such as betweenness centrality, degree of nodes, edge betweenness, closeness centrality, clustering coefficient, and others to present a condensed and robust nature of the components with each other in both the networks.

#### 3.4.1 Identification of top nodes present in M1/M2 network

**Figure 5.**
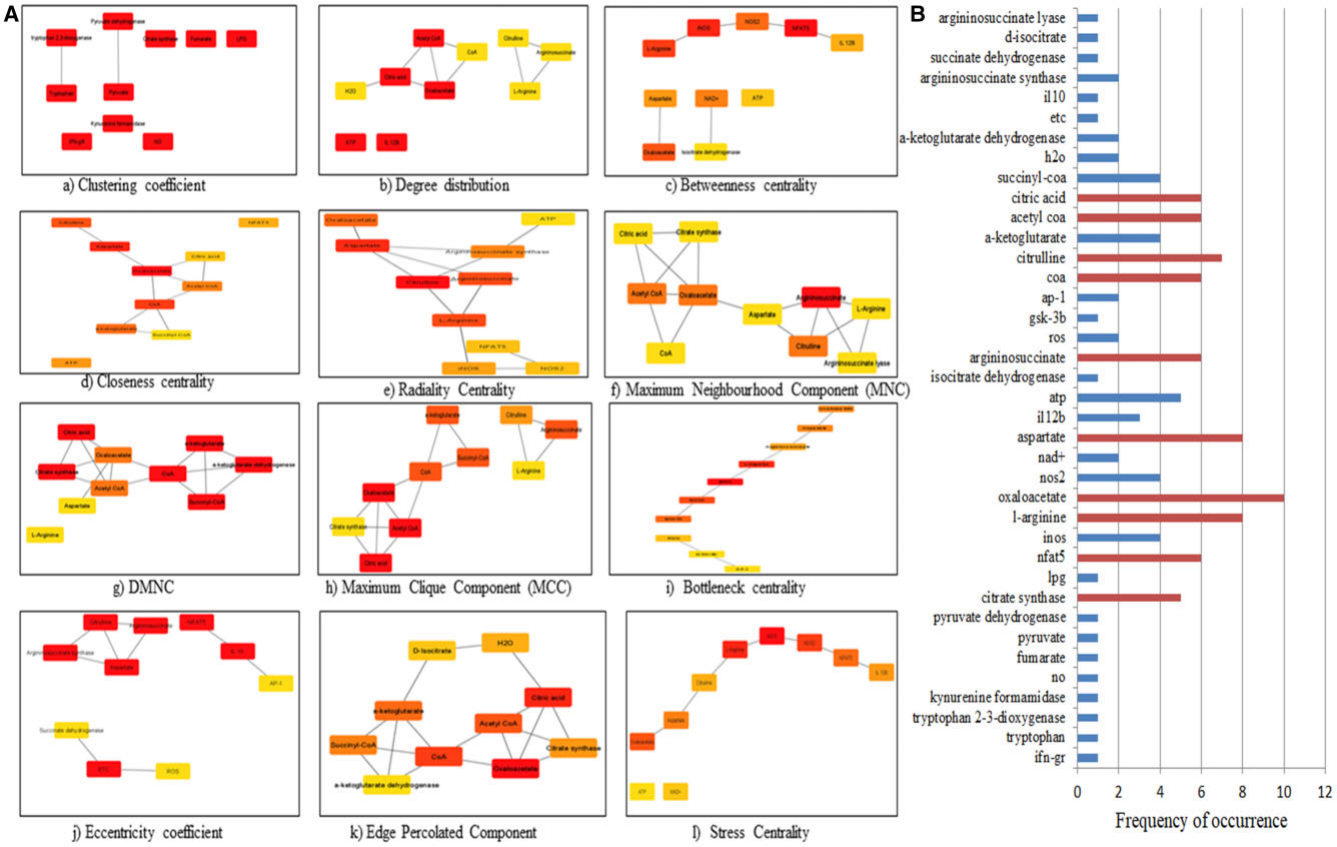
(A) For M1 network: Substantial modules based on clustering coefficient; degree distribution; betweenness centrality; closeness centrality; radiality; maximum neighbouring component (MNC); density maximum neighbouring component (DMNC); maximum clique centrality (MCC); bottleneck; eccentricity; edge percolated component (EPC); stress; were identified from the leading network. From top to bottom, red to light yellow in modules denotes the rank. (B) The top 10 ranked components in the M1 network based on their frequency of occurrence in the 12-scoring methods of Cytohubba are graphically represented.

The Cytohubba was used to identify the top-ranked nodes in the entire network based on each topological analysis methods. MCC, DMNC, MNC, EPC, radiality, degree, closeness, betweenness, eccentricity, bottleneck, clustering coefficient, and stress centrality are the 12 scoring methods that were used to identify top-ranked nodes in the network (Fig. 5A).

Each method yielded the top 10 nodes in the network. Based on their occurrence in each of the 12 scoring methods, the top 10 components are represented in Fig. 5B. Oxaloacetate was the most prominent in the M1 network analysis.

**Figure 6.**
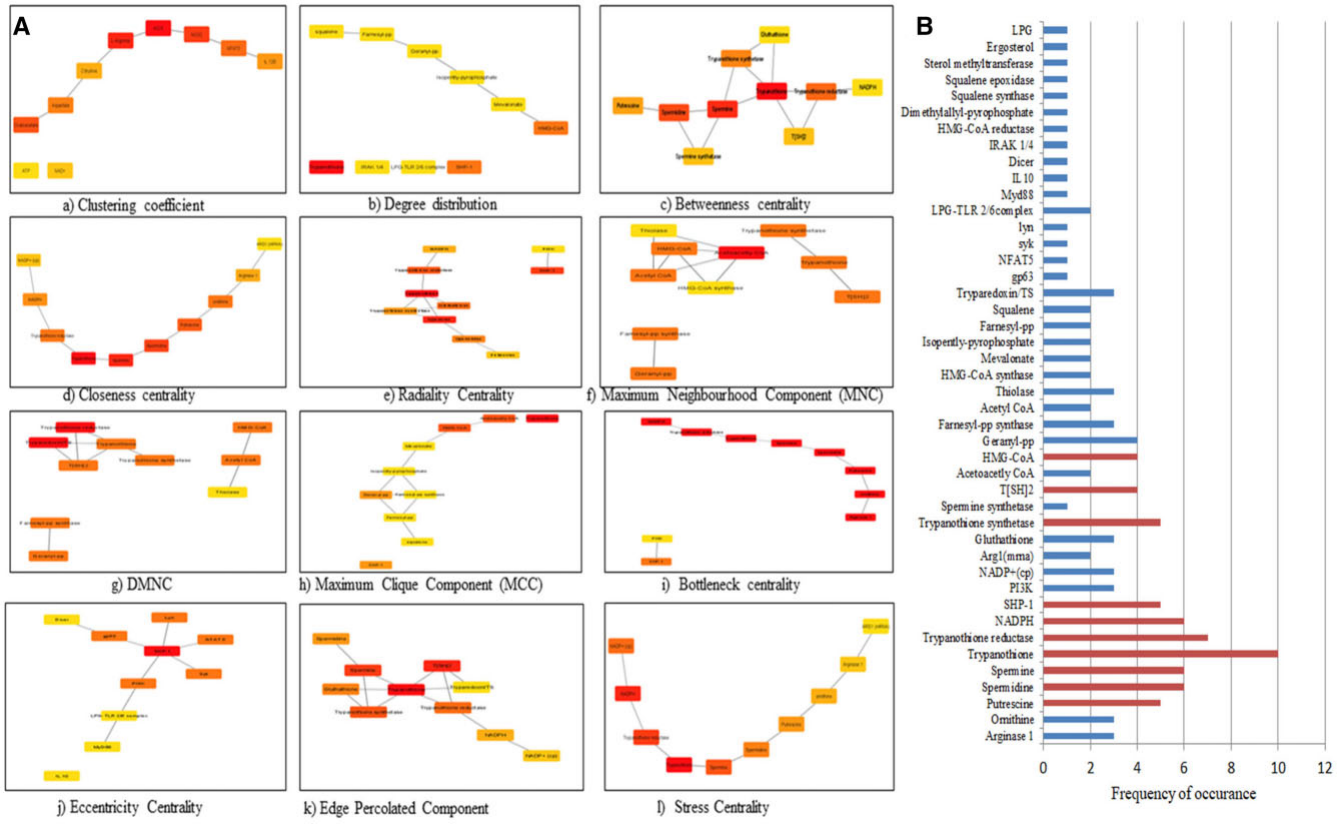
(A) For M2 network: Substantial modules based on clustering coefficient; degree distribution; betweenness centrality; closeness centrality; radiality; maximum neighbouring component (MNC); density maximum neighbouring component (DMNC); maximum clique centrality (MCC); bottleneck; eccentricity; edge percolated component (EPC); stress; were identified from the leading network. From top to bottom, red to light yellow in modules denotes the rank. (B) The top 10 ranked components in M2 network based on their frequency of occurrence in the 12-scoring methods of Cytohubba are graphically represented.

Based on the occurrence of nodes in each of the 12 scoring methods, the top 10 components are represented in Fig. 6A and B). Trypanothione was the most prominent in the M2 network analysis. SHP-1 was the only component from the host which was identified as a crucial node.

**Figure 7.**
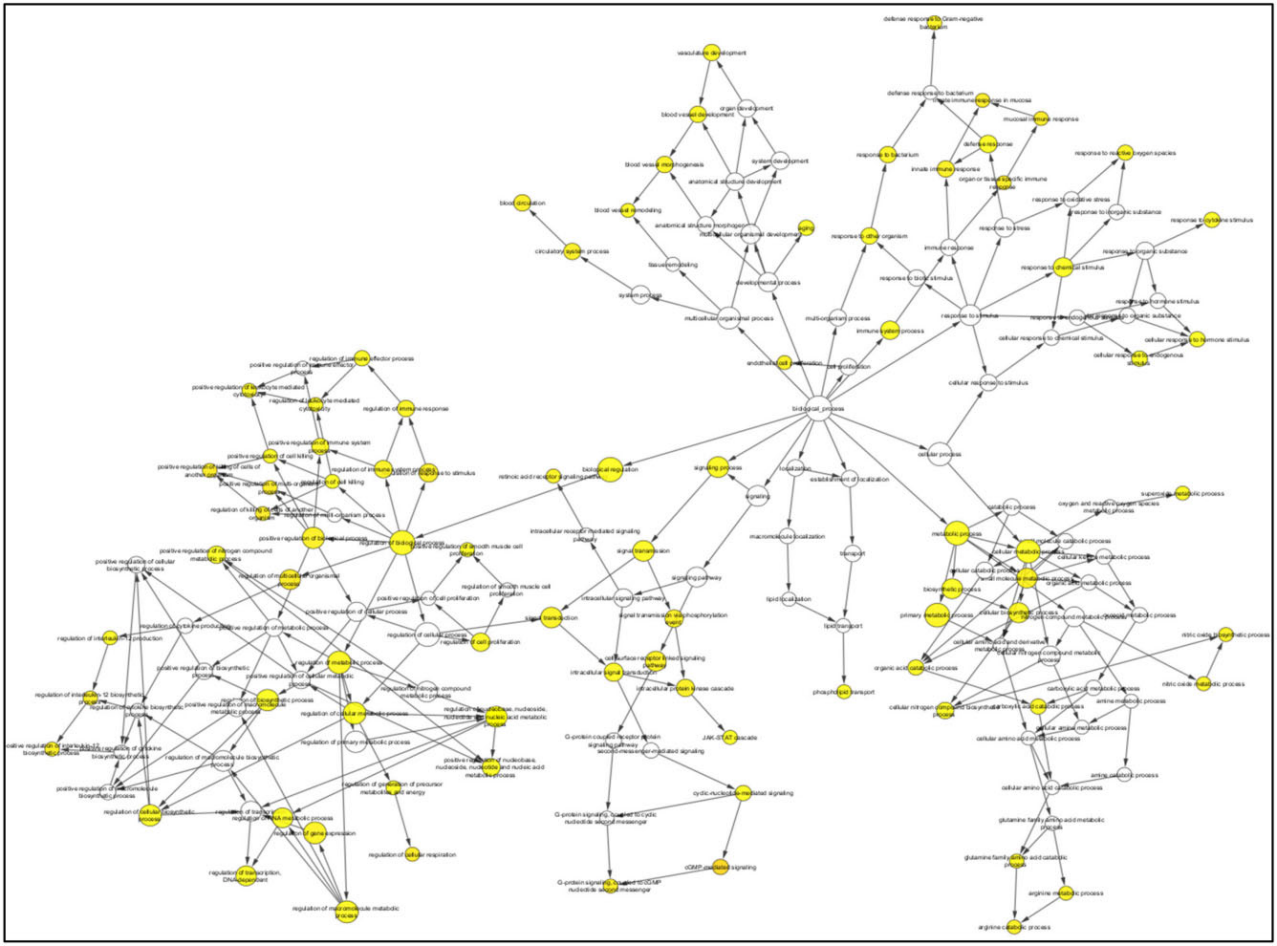
BiNGO visualization of identified components from M1 network (*P*-value 0.01).

The BiNGO, version 3.0.5, plugin was used for identifying gene ontology (GO) categories that were substantially over-represented in a set of genes-metabolites or a biological network sub-graph of M1 network and M2 network. We investigated and displayed the biological functions and cellular elements of discovered components of M1 network. Arginine metabolic process, glutamine family amino acid catabolic process, nitric oxide biosynthesis process, superoxide metabolic process, response to cytokine stimulus, regulation of IL-12 production, regulation of transcription, and regulation of cellular respiration (Fig. 7).

**Figure 8.**
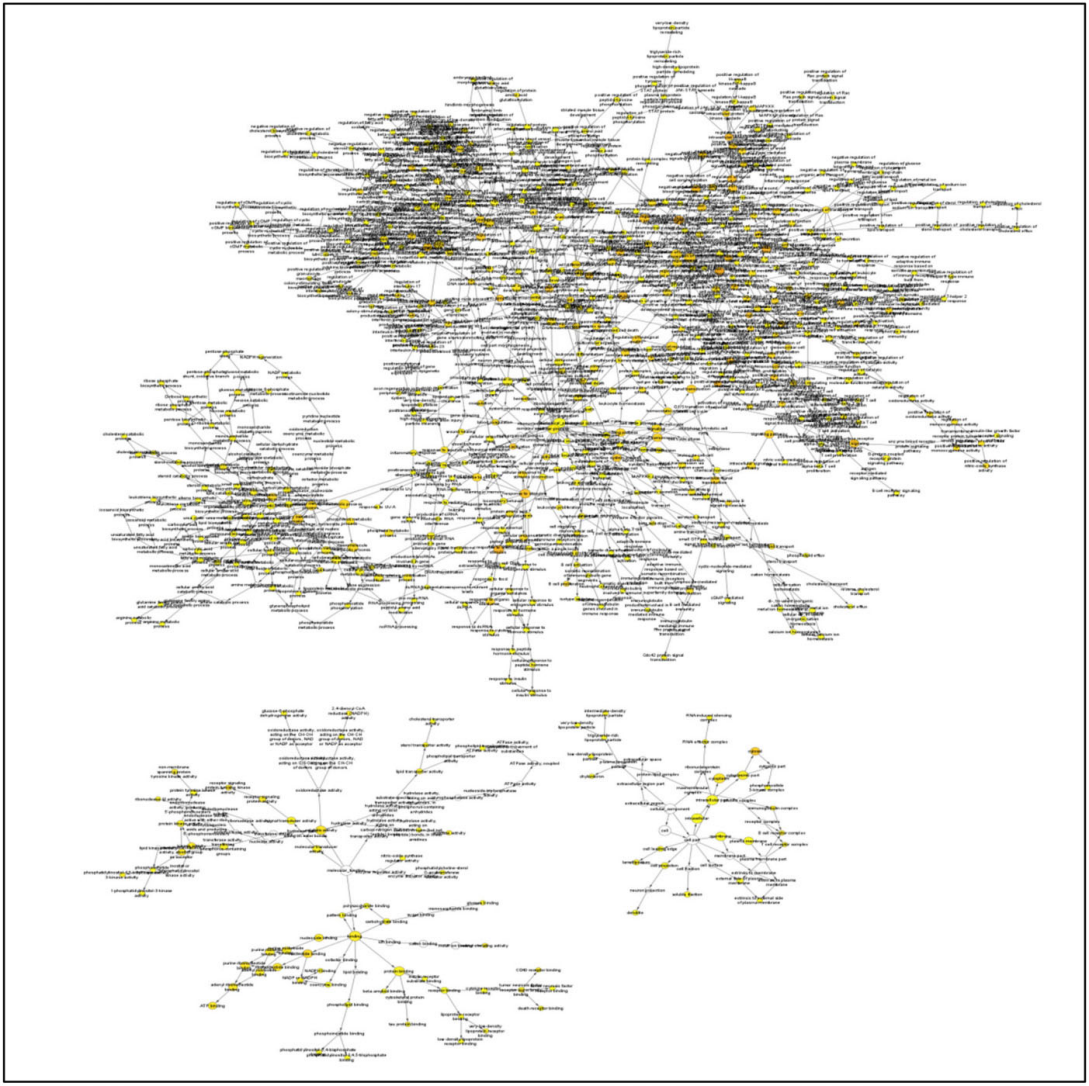
BiNGO visualization of identified components from M2 network (*P*-value 0.01).

The BiNGO, version 3.0.5, was used for GO categories for immune-metabolic biological network sub-graph of M2 network. We investigated and displayed ontologies associated with M2 components which highlighted significant involvement in cytokine signaling immune system, arginase activity, regulation of host lipid pathways, regulation of ROS, negative regulation of animo acid glutathionlylation, decreased IL-12 signaling, decreased NO production, and increased IL10 production (Fig. 8). Gene list for M1 and M2 associated gene ontology analysis is provided in Supplementary data (S6, S9, and S10).

## 4 Discussion

Immuno-metabolic response of host macrophages may decide fate of parasite infection; it may either promote parasite elimination or parasite survival. The extent of infection is dependent on multiple factors, including the species involved, host genetics, early innate immune response, adaptive immune response and crucially, the polarization of macrophages (Martínez-López *et al.* 2018). Macrophage polarization is known to be closely related with changes in metabolic signaling pathways and cellular metabolism, which are responsible for many essential macrophage functions like phagocytosis, motility, and mediator production (NO, ROS, cytokines, chemokines) (Wilson *et al.* 2019). The already available drugs primarily target metabolic process of parasite and recently many investigative drugs and vaccines are targeting immune response. Hence, we suggested, as both the pathways are interlinked to generate responses to disease, we should study them as a consortium of pathways interlinking immune-metabolic pathways in a holistic manner.

Through our M1/healthy state mathematical model, we observed that NO was high due to the high level of IL-12 and NFAT5 in the initial response, but as time progressed, the parasite promotes changes in the macrophage which hampers NFAT5 expression and promotes SHP-1 expression. The result from M2/diseased state mathematical model simulation analysis suggests that the levels of SHP-1 increases and NFAT5 decreases, which changes the phenotype of macrophage affecting IL-12 and NO concentrations. This may affect parasite clearance. SHP-1 activation prevents NO production by dephosphorylating NFAT5 which leads to change in utilization L-arginine (precursor of NO). As NO production is inhibited, L-arginine is shown to be used for the production of polyamines like spermidine in the presence of Arg1 which is synthesized through IL-4 mediated PPAR-γ activation. Spermidine is one of the precursors for trypanothione synthesis. Since NFAT5 is dephosphorylated, it may not bind to IL10 promotor, leading to activation of IL-10 synthesis.

Flux analysis defines the flow of molecules at a specific time (Beguerisse-Díaz *et al.* 2018). In M1 state, we observed that the reactions that are promoting IL-12, IFNγ, NO, and ROS production are high (flux, Table 3), which promotes parasite elimination. In M2 state, the reaction that is promoting SHP-1 activation, IL-10, IL-4, ergosterol, and trypanothione production had high flux, which indicates parasite growth and proliferation.

Through PCA we identified that both the dataset may be essential. From variable plot, the inverse correlation between M1 and M2 models signifies essentiality of components from both the datasets as cos2 value of both the datasets is same. We distributed the PCs based on significance of their concentration and identified 12 components for further studies. SHP-1 was the principal component from M2 dataset as it is directly activated by GP63 to inhibit NFAT5 through phosphorylating it at TYR143 residue thereby inhibiting its localization to nucleus affecting production of NO, IFNγ, and IL-12 in M1 model (Zhou *et al.* 2014, Tellechea *et al.* 2018). Further, IL-12 and IL-10 are principal components that are of opposite nature; their secretion will directly affect the parasite survival (Kane and Mosser, 2001, Park *et al.* 2002). AKT activation leads to GSK-3b inhibition (Patel and Werstuck 2021). GSK-3b negatively regulates IL-10 production and its inhibition can promote disease pathogenesis (Nandan *et al.* 2012). ADP is also a principal component as also the activation required ATP as an energy source (Carthew 2021). Impaired TCA cycle resulted in accumulation of succinate and increased ROS as a result of reverse electron transport in the respiratory chain (Saunders and McConville 2020). Ergosterol present in parasite acted as the first line of defence against the toxic effects of parasite eliminating molecules (Sannigrahi *et al.* 2017). Trypanothione act as a prime detoxifying molecule in parasite, thereby enhancing neutralization of ROS produced by host macrophages during the infection (Turcano *et al.* 2018).

The purpose of the network analysis was to look into the important components in terms of connectivity and its functioning present in both the network. For the M1 network, the top 10 components were oxaloacetate, L-arginine, aspartate, NFAT5, citrulline, citrate synthase, arginosuccinate, CoA, acetyl CoA, and citric acid. Oxaloacetate had highest frequency of occurrence score, which is one of the initial components for the impaired TCA cycle that later leads to the production of ROS. Other than oxaloacetate, acetyl CoA, citrate synthase, CoA, and citric acid were also part of the same impaired TCA cycle, which is important for ROS production (Saunders and McConville 2020). Furthermore, citrulline and arginosuccinate are byproducts of NO production as well as they are recycled for L-arginine synthesis which can be later directed again to NO production (Rath *et al.* 2014). NFAT5 functions are already signified with cytokine signaling and parasite elimination. It also shows inhibitory effects on IL10.

For the M2 network, the top 10 components were trypanothione, trypanothione reductase, spermidine, spermine, putrescine, trypanothione synthase, HMG-CoA, NADPH, T[SH]2, and SHP-1. Trypanothione had highest frequency of occurance score that crucially helps in parasite survival by neutralizing the ROS. Trypanothione synthase, NADPH, T[SH]2, trypanothione reductase are all responsible for continuous production of trypanothione. Spermidine and spermine are polyamines required for parasite proliferation and spermidine also acts as one of the initial components for trypanothione synthesis. Furthermore, HMG-CoA is intermediate for ergosterol production that strengthens the parasite cell membrane. SHP-1 is the only host component that was enriched in Cytohubba analysis. SHP-1 functions are associated with inhibition of pro-inflammatory cytokine synthesis through transcription factor inhibition that enhances IL-10 production, which helps the parasite in its survival. Hence, from our model we identified that SHP-1 has role in promoting and inhibiting different processes, which promote parasite survival.

## 5 Conclusion

Through our model and network analysis, we could infer that SHP-1, (a host factor) may promote parasite survival. This also suggests that parasite hijacks host machinery to regulate host associated transcription factors through chromatin remodeling. Thus, SHP-1 can be taken as a potential candidate for further studies to investigate parasite survival mechanism.

As a conclusion, we assert that computational modeling and network construction of immuno-metabolic response against leishmaniasis helps us to understand the signaling mechanism responsible for parasite survival. Thus, system’s biology approach gave us the necessary lead in a specific reconstructed biological network. By regulating SHP-1 through synthetic biology approach we might be able to find a better therapeutic for treating leishmaniasis by gaining better control over immune-metabolic signaling of host.

Our study has laid an insight into inflammation and macrophage associated infectious disease that is leishmaniasis. Such approach can be useful to study diseases which show dynamics in parallel with infection and inflammation, where cytokine expression and host pathogen metabolism might regulate the disease.

## Supplementary Material

vbad125_Supplementary_Data

## Data Availability

S1- Total flux of M1 model; S2- Total flux of M2 model; S3- PCA matrix; S4- M1 network file; S5- M2 network file; S6- Gene list for Gene ontology; S7- Initial concentration of M1 components; S8- Initial concentration of M2 components; S9- Gene ontology analysis of M1 model; S10- Gene ontology analysis of M2 model.
